# Keeping reptiles as pets in Brazil: keepers’ motivations and husbandry practices

**DOI:** 10.1186/s13002-023-00618-z

**Published:** 2023-10-21

**Authors:** María Fernanda De la Fuente, Bruna Monielly Carvalho de Araújo, Iamara da Silva Policarpo, Heliene Mota Pereira, Anna Karolina Martins Borges, Washington Luiz Silva Vieira, Gentil Alves Pereira Filho, Rômulo Romeu Nóbrega Alves

**Affiliations:** 1 Programa de Pós-Graduação em Etnobiologia e Conservação da Natureza, Universidade Estadual da Paraíba, Campina Grande, PB Brazil; 2 Programa de Pós-Graduação em Ciências Biológicas (Zoologia), Universidade Federal da Paraíba, João Pessoa, Brazil; 3 LAPEC - Laboratório de Peixes e Conservação Marinha, Universidade Estadual da Paraíba, João Pessoa, Brazil; 4 Programa de Pós-Graduação em Ciências Biológicas (Zoologia), Laboratório de Ecofisiologia Animal, Departamento de Sistemática e Ecologia, Centro de Ciências Exatas e da Natureza, Universidade Federal da Paraíba, João Pessoa, Brazil; 5 Departamento de Biologia, Universidade Estadual da Paraíba, Campina Grande, PB Brazil

**Keywords:** Ethnozoology, Animal welfare, Companion animals, Exotic pet, Netography

## Abstract

**Background:**

Reptiles are considered one of the most popular pets in the world and are often associated with an incorrect belief that they are simple, highly adaptable, and easy to keep animals when compared with other pets, such as dogs and cats. However, keeping reptiles as pets can pose several challenges in meeting their needs in a domestic setting, requiring specific conditions and effort to maintain their health, well-being, and survival.

**Methods:**

During 2015, using online semi-structured questionnaires applied to 719 Brazilian pet reptile keepers who participated in online groups of reptile breeders on the social network Facebook, this study aimed to identify Brazilian keepers’ motivations for maintaining reptiles as pets, investigate their monthly expenses, and the husbandry practices for the maintenance, such as housing and feeding conditions, handling of the animal, health issues, and treatment provided.

**Results:**

We found multiple motivations for keeping reptiles as pets (mostly snakes, lizards, and chelonians), the main motivation being emotional reasons, followed by entertainment and convenience reasons. The great majority of keepers (69%) declared to spend less than or up to US$30 per month in maintaining their reptiles. Most reptiles were kept alone in terrarium/aquarium enclosures, with basic environmental complexity in terms of physical elements. Lizards and chelonians were fed with a few insect species, cultivated fruits and vegetables, while snakes were fed mainly with domestic rodents, rabbits, or birds. Keepers declared frequent cleaning of the enclosure, but inappropriately handled their animals directly with their hands, which might result in potential threats to human and reptile health and safety. Several diseases or injuries were mentioned and 55.6% of the keepers declared taking the reptile to the vet for treatment.

**Conclusions:**

Overall, our findings revealed several challenges that reptiles face when kept in domestic environments, including issues related to housing, nutrition, and healthcare. Even though keepers demonstrated positive feelings toward their pets, suggesting a positive relationship and a willingness to provide them with proper care, it seems that without the proper knowledge and awareness, reptiles may unintentionally be kept with poor husbandry. Addressing these challenges on husbandry practices is essential for improving reptiles’ welfare and promoting a responsible pet ownership.

## Background

Exotic pets are non-traditional or uncommon animals kept as pets, they can be non-native or native to a region, not domesticated, and include a wide range of animals from several taxonomic groups, such as birds, primates, reptiles, small mammals, amphibians, invertebrates, and fishes [1, 2]. Among them, reptiles are considered one of the most popular pets in the world [3–5] and its global market has grown significantly in the last decades [6, 7]. Reptiles’ popularity as pets is often associated with an incorrect belief that they are simple, highly adaptable, and easy to keep animals, perfect for people who might not have time, resources, or energy to care for other types of pets that require more attention (e.g., dogs and cats) [8]. However, keeping reptiles as pets can pose several challenges and difficulties in meeting their needs in a domestic setting.

Studies have shown that reptiles are more cognitively [9–11] and socially [12, 13] complex than previously thought, requiring specific conditions and considerable effort to maintain their health, well-being, and survival [8]. Different reptile species have evolved specific social, behavioral, and ecological needs that are often not satisfied in captive situations. For example, several reptile species are very active and move throughout the natural environment in different ways (e.g., climbing, walking, running, swimming, burrowing), to search for food, mates, evade predators, defend their territories, socialize, and engage in other complex movements and behaviors [14–17]. However, the poor-quality information supplied at the time of purchase, and the overall lack of knowledge about the biology and natural lifestyles of many reptile species results in poor husbandry practices that fail to meet their basic requirements, causing distress, suffering, and illness [18, 19].

Inappropriate husbandry conditions, such as maintaining individuals in small and barren enclosures, with incorrect temperature and humidity levels, a poor diet and nutrition, as well as inadequate hygiene, handling, and health treatment are some of the most common issues reptiles face when kept as pets [8, 20]. Subjecting animals to negative experiences (e.g., hunger, fear, inability to express normal behavior, pain) contributes to poor animal welfare, health problems, and eventually death [21–23]. In general, humans do not have the ability to perceive or assess reptiles’ welfare status, as most reptiles do not communicate these aspects through facial expressions, body language, vocalizations, or clear-cut behaviors, and usually hide signs of illnesses [19, 24]. The difficulty in recognizing distress and suffering in most reptile species aggravates the state of pet reptiles’ welfare and mortality rates, especially for less common and more fragile species.

The pet trade regulation in Brazil is currently guided by poorly implemented policies. The main problems are associated with failures in legislation and enforcement, corruption, and lack of resources [25]. In Brazil, it is prohibited the import of any exotic reptiles for use as pets, and the ownership and trade of exotic animals are regulated by IBAMA (Brazilian Institute of Environment and Renewable Natural Resources) is illegal to breed, capture or trade any wildlife species without its authorization. However, regardless of legal restrictions, several reptile species, both native and exotic, endangered, or not, have been kept throughout the country for the purpose of companionship [4, 26, 27]. Some Brazilian states have independently published their regulations and species lists with different and even opposite positions. For example, in the case of reptiles, while the state of Rio de Janeiro prohibits the establishment of commercial breeding grounds for exotic animals, the state of Alagoas regulated this practice [25].

Investigating why and how reptiles are kept as pets is important to better understand this practice and improve pet reptiles’ welfare by detecting potential husbandry and care problems and deficiencies. This paper is a continuation of the study on different aspects of keeping reptiles as pets in Brazil. Here, we aimed to identify Brazilian keepers’ motivations for maintaining reptiles as pets, investigate their monthly expenses, and the husbandry practices for the maintenance. Specifically, we examined the housing/enclosure conditions (type, complexity, number of cohabitants, and hygiene), diet and feeding frequency, handling of the animal, health issues, and treatment provided. We hypothesize that (1) the biggest motivation to keep a pet reptile is the convenience due to low-maintenance misconception; (2) monthly expenses spent on reptile care and husbandry are overall low; and (3) most reptiles are kept in enclosures with low complexity conditions. Finally, we discuss the implications of keeping reptiles as pets for their animal welfare and species conservation.

## Methods

### Data collection

From January to November 2015, the authors obtained the data using online semi-structured questionnaires applied to 719 pet reptile keepers in Brazil who participated in 29 online groups of reptile breeders and sales/trade on the social network Facebook (see Alves et al. [4] for details on the search method). We made initial contact with each breeder via private message to invite them to participate in the research, explain its aims, and present them with the Informed Consent Form, requesting their signature as formal consent to participate in the study. Once the invitation to participate was accepted and the form signed, we sent a new message with the link to the online questionnaire (Google Forms platform). Information regarding specific reptile species kept, their conservation status, and the socioeconomic profiles of keepers (age, sex, religion, marital status, educational level, and family income) are published in a separate publication [4]. Here, we present data on the keepers’ motivations to keep reptiles as pets; monthly expenses with the animal(s); and the conditions of husbandry and maintenance including enclosure type and complexity, number of co-habitants in the enclosure, diet, feeding frequency, handling of the animal(s), frequency of enclosure hygiene, health issues and treatment provided. The study was approved by the Research Ethics Committee of Hospital Universitário Lauro Wanderley (CEP/HULW, authorization # 853.115).

### Data analysis

We used descriptive statistics to describe keepers’ responses related to the conditions of husbandry and maintenance of pet reptiles. We calculated and report the frequencies and percentages based on the total number of interviewees (n = 719). Since almost half of the keepers (n = 334) owned more than one specimen of the same or different species (971 specimens from 69 species, [4]), the sum and percentages may exceed the total number of interviewees depending on each question and keepers’ responses.

To test our hypotheses, we conducted Chi-square Goodness of Fit analyses to compare the observed frequencies of (1) motivations to keep a pet reptile; (2) monthly expenses spent on reptile care and husbandry; and (3) the enclosure complexity that reptiles are kept in, versus the expected frequencies (i.e., frequencies in each category of categorical variables are equal: null hypothesis). We divided the motivation variable into six categories taking into account keepers’ responses: “emotional reasons” (keepers used terms that transmitted admiration, love, affection, like, fascination, passion, among others); “convenience” (keepers reported that reptiles are easy to maintain, cheap, or have advantages over other pets such as fur allergy, longevity); “entertainment” (keepers kept reptiles as a hobby, curiosity, enjoy their exotic beauty and behavior); “educational purposes” (some keepers were biologists, used them for educational activities, and to learn about behavior/ecology); “accidental owners” (the reptile was rescued or a gift); and “conservation” (to preserve the species). We divided the enclosure complexity into five categories: “barren” the space is sterile with no elements on it or with only artificial substrate (e.g., newspaper, kitchen paper); “basic elements” the space has few elements such as natural substrate and/or a log or stone; “three-dimensional” besides natural substrate, the space has several elements such as plants, logs, rocks, platforms, refuges or hiding places, providing several options of vertical space, climbing facilities, choices for perching, visual barriers, shelter and privacy areas, allowing the animal opportunities to remove themselves from the view of humans or cage-mates/cohabitants; “adequate” besides elements cited in the previous category, the space counts with a heat or light source and water or misting features other than bowl of drinking water, we assume that this provide the animal(s) with proper environmental conditions (humidity, lighting, and temperature); and “free roam” animals are not restricted to an enclosure, they can move freely in the space in which they are kept, having a number of opportunities to choose where they want to be an how they want to behave. Finally, we divided the monthly expenses variable into seven categories: “US$0”, “ ≤ US$30”, “US$30–60”, “US$61–90”, “US$91–150”, “ > US$150”, and “do not know”.

We used a word cloud analysis to further evaluate the keepers’ motivations to keep reptiles as pets. This analysis makes it possible to visually identify the most frequently cited words by the keepers [28]. We conducted all analyses using R studio software, version 3.6.2. For all analyses, we set the statistical significance level at 0.05.

## Results

**Fig. 1:**
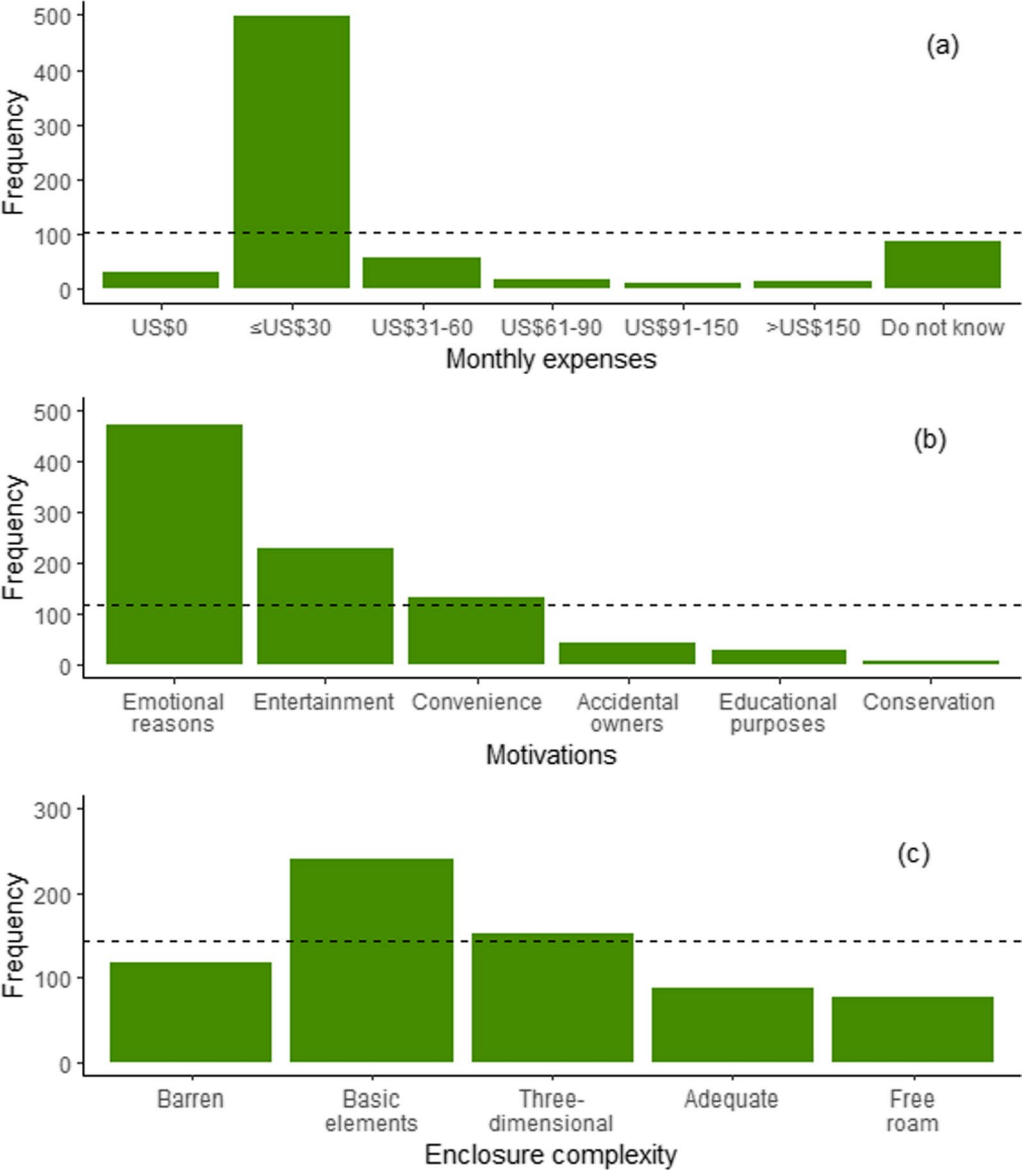
Observed frequencies of **a** monthly expenses for keeping pet reptiles; **b** motivations for keeping pet reptiles; and **c** enclosure complexity. Dashed lines indicate the expected frequency values for each variable (i.e., all categories are equal: null hypothesis)

**Fig. 2:**
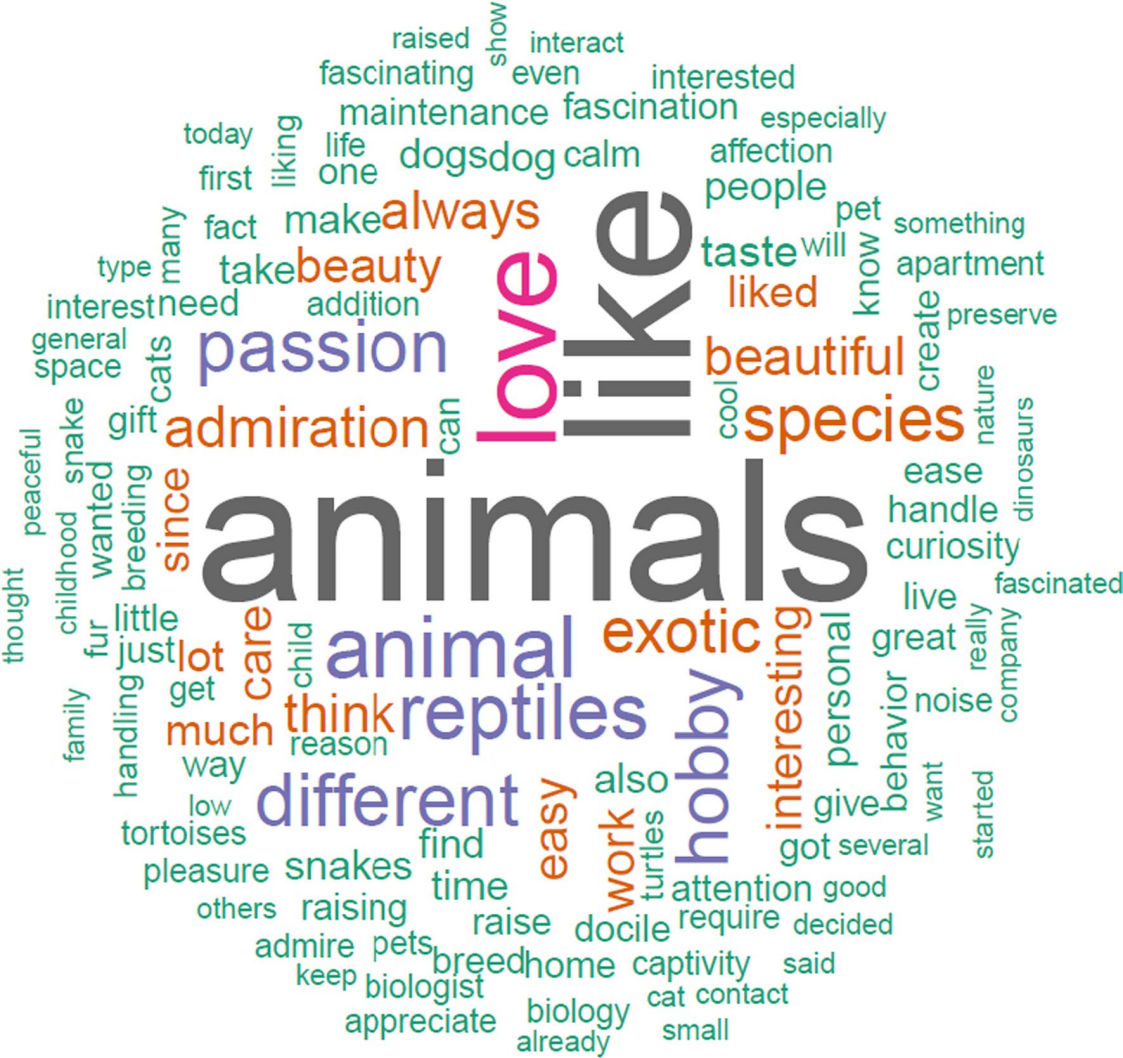
Word cloud of the most 100 frequent words from keepers’ responses (n = 719) regarding their motivation for keeping pet reptiles. Word sizes indicates the frequency of citation, the larger the word the more frequently it was cited

Of the 719 keepers interviewed, 67 kept more than one reptile group. More than half of the keepers (n = 410, 57%) kept snakes, 233 of them (32.4%) kept chelonians, 142 (19.7%) kept lizards, and only one keeper (0.14%) kept a crocodilian specimen. Most keepers (n = 358, 49.8%) kept only one reptile specimen, followed by two (n = 147, 20.4%) and three (n = 66, 9.2%) specimens (mean ± SD = 3.15 ± 7.5 specimens; range from none reptile kept at the time of the interview (n = 22, 3%) to > 100 specimens (n = 2, 0.3%)). The great majority of keepers (n = 497, 69%) declared to spend up to US$ 30 monthly to keep their reptiles (*X*^2^ = 1810.8, *df* = 6, *p* < 0.0001, Fig. 1a). Regarding the motivations for keeping a pet reptile, 215 keepers (30%) declared more than one motivation. Most keepers (n = 472, 65.6%) declared they keep pet reptiles because of emotional reasons, 229 keepers (32%) maintain reptiles as pets for entertainment, 131 keep reptiles (18.2%) because of convenience, 42 keepers (6%) were accidental owners, while 29 (4%) keep them for educational purposes, and 8 (1.1%) for conservation (*X*^2^ = 1032.3, *df* = 5, *p* < 0.0001, Fig. 1b). Keepers described their motivations for keeping pet reptiles using several words, the most cited were “like”, “animals”, “love”, “passion”, “different”, “hobby”, “admiration”, “beautiful”, “exotic”, “interesting”, “easy”, “beauty”, among others (Fig. 2).

**Fig. 3:**
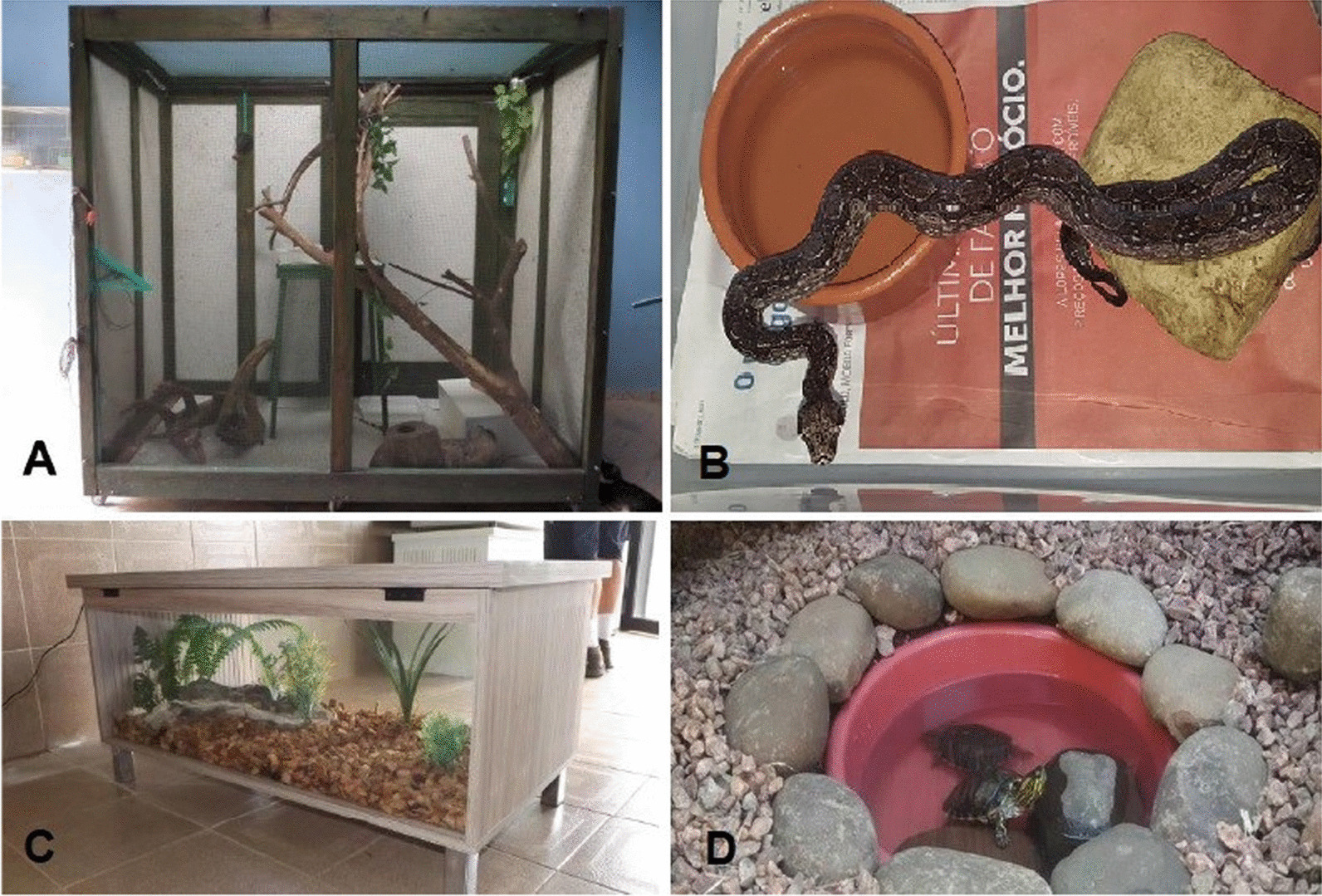
Examples of types of enclosures used by pet reptile keepers. **A** Cage; **B** *Boa constrictor* housed in a plastic box; **C** Terrarium; and **D** *Trachemys dorbigni* kept outdoors in the keeper’s backyard

Keepers described six types of enclosures or environments used by pet reptiles’ keepers. Terrarium/aquarium: enclosure made of glass supported by iron or wooden base; Cage: enclosure made of wire mesh supported by an iron or wooden base; Box: made of cardboard or plastic container; Indoors free roam: reared loose inside the keeper’s home; and Outdoors free roam: reared loose in the keeper’s backyard (Fig. 3). Most snakes (n = 367, 89.5%) and lizards (n = 111, 78%) were kept on terrariums/aquariums, while most chelonians were housed on terrariums/aquariums (n = 109, 46.8%) or kept outdoors free roam (n = 88, 37.7%). The only crocodilian was kept in a terrarium (Table 1A). Regarding enclosure complexity, most keepers (n = 241, 33.5%) declared to add basic elements (natural substrate and/or a log or stone) in their reptile enclosure, followed by those (n = 153, 21.3%) who added several elements enhancing a three-dimensional environment, and those who (n = 118, 16.4%) kept their reptiles in a barren enclosure. Fewer keepers (n = 87, 12.1%) maintain an adequate environment including a heat/light source and water/misting features other than drinking water (*X*^2^ = 129.56, *df* = 4, *p* < 0.0001, Fig. 1c). 77 (10.7%) keepers declared to keep their reptiles free roam and 43 (6%) keepers did not answer this question. Table 1B contains detailed information on enclosure complexity per reptile group. Keepers declared that most specimens were housed alone (Table 1C). Snakes were housed with up to three specimens, lizards with up to four specimens, and chelonians with up to eight specimens of the same species (Table 1C).

**Table 1:** Frequency of keepers’ responses regarding husbandry practices (enclosure type and complexity, co-habitants, feeding frequency, and handling) per reptile group (snakes, lizards, chelonians, crocodilians)

		Reptile group			
		Snakes	Lizards	Chelonians	Crocodilian
A. Enclosure type	Terrarium/aquarium	367	111	109	1
	Cage	26	17	24	–
	Box	14	4	1	–
	Outdoors	0	6	88	–
	Indoors	3	4	9	–
B. Enclosure complexity	Barren	61	15	47	–
	Basic elements	140	57	76	–
	Three-dimensional	109	35	28	1
	Adequate	69	19	5	–
	Free roam	1	7	69	–
C. Co-habitants	Alone	307	73	117	1
	+ 1	17	17	40	–
	+ 2	4	6	18	–
	+ 3	0	1	6	–
	+ 4 to + 7	0	0	6	–
D. Feeding frequency	More than once a day	0	22	65	–
	Daily	1	71	131	1
	More than once a week	29	24	17	–
	Weekly	182	6	0	–
	Once every 10 days	57	0	0	–
	Biweekly	81	0	0	–
	Monthly	23	0	0	–
E. Handling	Hands	360	133	225	1
	Hooks	36	0	0	–
	Gloves	12	3	6	–
	Does not handle	1	6	1	–

Diet varied among the different reptile groups. According to the keepers’ responses, snakes were exclusively fed with alive or slaughtered whole prey, including rodents (*Mus musculus*), rabbits (*Oryctolagus cuniculus*), and small birds (quail and chicks, species not specified). Lizards and chelonians were fed with omnivorous diets that could include a mix of varied food items. For lizards, the diets mainly consisted of alive or slaughtered whole prey (rodents, small birds), insects (mealworms, cockroaches, crickets), vegetables, fruits, eggs, and commercial feed. For chelonians, diets consisted of whole prey (fish, shrimps), meat (beef and chicken), commercial feed, dehydrated shrimps (for chelonians), eggs, vegetables, and fruits. Only nine (1.3%) keepers specifically mentioned including vitamin supplements in their reptiles’ diets. Feeding frequency varied among and within each reptile group (Table 1D). Snakes were fed mostly once a week or more, some keepers specified that this occurred after complete digestion from the previous prey (after defecation). Lizards and chelonians were mostly fed daily or more than once a day. The only crocodilian was fed daily with whole prey (rodents and small birds). 55 keepers did not answer this question.

The majority of keepers declared that they hygienized the reptile enclosure on a weekly basis (n = 195, 27%), on a daily basis (n = 114, 15.8%), or as required (i.e., not specified, keepers responded that they cleaned the enclosure as soon as it got dirty, n = 115, 16%). Regarding the handling of all reptile groups, most keepers directly use their hands, without gloves, to handle the reptiles (Table 1E). Only 88 keepers (12.2%) mentioned that their reptile had ever become sick/ill. Reptile diseases or injuries cited were pneumonia (n = 14), stomatitis (n = 10), worms (n = 7), diarrhea (n = 4), fungus (n = 4), prolapse (n = 3), colds (n = 3), tumor (n = 3), wounds (n = 3), accidents (n = 2), and dehydration (n = 2). Other illnesses mentioned (n = 1) were foreign body ingestion, apathy, bronchitis, intestinal calcification, dermatitis, scaling difficulty, dystocia, blister disease, calcium deficiency, gingivitis, retained eggs, pellagra, and virus. For treatment, 49 keepers (55.6%) took the reptile to the vet, 26 (29.5%) medicated or managed the reptile environment (e.g., adjusted the feeding, humidity, or temperature of the enclosure) without a vet indication, 2 keepers (2.3%) applied home treatment, 2 keepers (2.3%) took the reptile to another breeder for advice, and 9 keepers (10.3%) did not do anything at all.

## Discussion

In the present study, we found multiple motivations for keeping reptiles as pets among Brazilian keepers, the biggest one being emotional reasons (e.g., like, love, admiration), followed by entertainment and convenience reasons as also dominant/important motivations. Because of the overall misperception that reptiles are easy to keep [8], we expected convenience to be more prevalent than other motivations for keeping pet reptiles, but this was not the case. It seems that affection for animals and the desire to be close to them is the main driver for pet-owning, regardless of the type of pet (i.e., domesticated or exotic species) [29]. Motivations were overall complex and multidimensional, with keepers having more than a single reason for keeping reptiles as pets. This finding is in agreement with other studies that have also shown affection and emotional relations as well as a variety of other motivations for keeping exotic pets [20, 29, 30]. For example, in Portugal, most keepers described affection toward reptiles to explain the acquisition and keeping of reptiles as pets, while convenience, entertainment, companionship, and duty of care were the motivations for long-term keeping [20]. In Greater Jakarta, Indonesia, the motivations for keeping snakes were mainly influenced by seeing close peers keeping snakes, visiting snake exhibitions, social media, or gifts [31]. In Russia, motivations to keep exotic pets were pity (“life-savers” keepers), looking for something different/unusual (“new experience seekers”), acquiring animals based on their specific characteristics (“collectors”), or by chance (“accidental owners”) [30]. In the United Kingdom, the motivations for having a pet reptile vary from media-based interest, wanting a reptile for a status symbol, wanting a pet, wanting a reptile since childhood, and interest in biology or natural history [32]. Differences in terms of the motivations of keeping reptiles or other exotic animals as pets might derive from cultural and social factors that affect the keeper’s knowledge, beliefs, desires, and their relationships with animals in general [29, 33]. Prokop et al. [34] assert that keeping pets results in emotional and cognitive experiences that can be generalized to even less popular animals such as snakes.

Overall, keepers demonstrated positive feelings toward their reptile pets, which might indicate a positive relationship with their animals and suggest a willingness to provide them with proper care, but might not always be related to proper knowledge, and pets may unintentionally be kept with poor husbandry. Even though reptiles were mostly kept by middle- and high-class individuals in terms of income [4], the great majority of Brazilian keepers declared to spend less than or up to US$30 per month in maintaining their reptiles. Besides the initial cost involved in the acquisition of the reptile that can be high depending on the species [4], many reptiles have specific requirements related to feeding and enclosures, resulting in continuous high maintenance costs [8]. Therefore, the low monthly expenses directed to this practice might reflect overall inadequate maintenance. It is important to note that the resources (i.e., money, time, and energy) required for keeping reptiles as pets can vary widely depending on the species, number, size, and lifespan of individuals. For example, snakes can be considered more “budget-friendly” (eating once a week or more), while other reptiles such as large lizards (e.g., tegu lizard, bearded dragon) can be more expensive to maintain, costing as much as a cat or a small dog [35, 36]. In this study, the main reptile group kept as pets were snakes and most keepers maintained only one specimen, which might explain the low monthly expenses found. The unanticipated large amount of money required for proper caring when acquiring an exotic animal (e.g., feeding, environment, specialized veterinary care) might result in animals being inadvertently neglected [30].

Most reptiles were kept alone in terrarium/aquarium enclosures, with basic environmental complexity in terms of physical elements. In the wild, reptiles live in complex physical three-dimensional habitats composed of abiotic (e.g., soil, rocks, temperature, lighting, water features) and biotic components (e.g., different plant and animals species); a temporal dimension (e.g., climate and resource variations in time); and a social dimension (e.g. presence of individuals of the same and different species) [37]. Nonetheless, for reptiles kept in captivity as pets, the need for stimulating and changing environments is often underestimated [38]. Except for some chelonians kept free roaming in gardens, keeping pet reptiles confined in closed enclosures is a common practice all over the world [19, 39]. This is often justified by the necessity of meeting specific temperature, humidity, and UV lighting requirements, as reptiles are ectothermic, relying on external sources of heat to regulate their body temperatures [20]. Specialized equipment such as heaters, heat lamps or pads, humidifiers, or misting systems, besides thermostats, thermometers, and hygrometers are needed to provide the appropriate environmental conditions. However, only 12% of the keepers included a heat/light source and a water/misting feature in their reptile’s enclosure, suggesting potential inadequate environmental comfort. Enclosures commonly found in households might also restrict environmental variation due to their small size, limiting animals’ ability to select between a range of thermal gradients and/or microhabitats along their activity period, resulting in less freedom of choice, less sense of control over their environment, less suitable physical and mental comfort, and poor welfare [40–43]. Furthermore, not providing the appropriate environmental conditions can lead to health problems, such as respiratory infections or metabolic issues [8, 41, 44].

Adding complexity to an enclosure (physical, social, environmental variation) is a form of environmental enrichment [37], and although it can be limited by the enclosure’s available space, its role is to create instances in which animals can express as many natural behaviors as possible. The presence of different substrates, vegetation cover, and physical structures such as burrows to afford shelter and retreat, rocks, perches, and other features to stimulate movement and exercise, can help individuals to reduce boredom, cope with the forced interaction of humans or other animals, and ultimately to thrive [45]. More complex enclosures result in more natural behaviors being displayed and more positive experiences, resulting in better animal welfare [37, 38]. A proper enclosure complexity should consider the species’ habits (e.g., terrestrial, arboreal, semi-terrestrial) and natural habitat (e.g., humid or arid environment) [8]. For example, terrestrial desert reptiles will benefit from sandy substrates, hiding caves, rocks, and lower branches to climb, relatively higher temperatures, and low humidity. On the other side, an arboreal rainforest reptile will prefer a moisture-retaining soil-based substrate, vertical space using branches and vines, foliage-covered areas, elevated water sources, higher humidity, and an adequate thermogradient. It is also important to consider the specimen characteristics (e.g., age, sex, size, how much it will grow, and social characteristics) to offer suitable space and adequate conditions [37]. For example, space that allows snakes to fully stretch to adopt rectilinear behavior/posture is important to their health and welfare [40]. In addition, while some reptiles may be solitary and be stressed by close proximity to cage mates, others might require higher levels of socialization [13, 46]. Overall, using different types of environmental enrichment (i.e., social, sensory, cognitive/occupational, physical, and nutritional) help to increase positive experiences that lead to a good mental state [47].

Even though enclosures should be functional for the animals, it seems that keepers might be prioritizing their needs over reptiles' needs in terms of facilitating cleaning and the handling of the animal. Indeed, frequent cleaning, as found here, is key to avoiding harmful microorganisms that can jeopardize health, such as bacteria, viruses, and parasites [8]. Nonetheless, we found that most keepers inappropriately handled their animals directly with their hands, which might result in potential threats to human and reptile health and safety (e.g., contagious diseases, accidents, and injuries). Reptiles have the potential of naturally carrying pathogenic organisms and can spread zoonotic diseases, such as salmonellosis, botulism, campylobacteriosis, and leptospirosis by direct contact with the animal or indirect contact with stool-contaminated surfaces or food [48]. Additionally, simple operations such as improper handling can be a strong stressor to reptiles and further contribute to the development of diseases [8]. Good sanitary conditions, safe handling, and proper veterinary care are effective ways to prevent concerns in animal health as well as public health risks. Therefore, besides considering the reptiles’ needs, habits, and characteristics to create environmental complexity and good welfare, keepers should also consider health and safety in their husbandry practices. A balance between good husbandry practices in terms of hygiene and handling and the biological and behavioral needs of reptiles should be maintained to create safe, functional enclosures for both animals and keepers.

Besides inadequate hygiene and handling, other diseases or injuries can result from an improper diet and nutrition. Different reptile species have specific dietary requirements that can be not fully known or complex and difficult to meet in captivity [22]. Usually, the provided diet does not stimulate natural feeding habits/behaviors and is not as balanced and varied as in nature, especially for reptiles with specialized diets. Moreover, the diet or nutritional needs of many reptile species have not been studied and knowledge is limited [49, 50]. Keepers fed lizards and chelonians with a mix of a few hand-raised insect species, and cultivated fruits and vegetables, among others, while in the wild, they can explore a diverse variety of foods [51, 52]. Similarly, snakes were fed mainly by domestic rodents, rabbits, or birds, which are easier to obtain, but in nature, snakes can prey on a wide range of species [52, 53]. Unbalanced diets can result in obesity or malnutrition, while vitamin or mineral deficiencies or overdoses might result in other illnesses such as metabolic bone disease (calcium deficiency), hypovitaminosis A (vitamin A imbalance/deficiency), or kidney disease (water deprivation, high-protein diet, excessive vitamin D supplementation) [52]. Very few keepers mentioned/highlighted supplementing reptiles' diets with vitamins or minerals when asked about their reptile’s diets. Since we did not specifically ask for supplementary vitamins on the diet, we should be cautious to interpret this result and further research should be conducted to better understand diets’ potential deficiencies and their consequences. Finally, qualified reptile veterinarians can be difficult to find, or proper medical care for reptiles can be more expensive (e.g., specialized knowledge, exams, and treatments), which can lead to keepers not seeking or obtaining proper treatment or relying on other non-specialized keepers or breeders to deal with complex health problems, resulting in animal suffering and potentially death. However, as discussed above, health issues and expensive veterinary costs can be avoided by providing pet reptiles a proper husbandry, diet, and environment.

## Conclusion

In conclusion, this study has shed light on the Brazilian keepers’ motivations for maintaining reptiles as pets and their husbandry practices. Our findings have highlighted several significant challenges that reptiles face when kept in domestic environments, including issues related to housing, nutrition, and healthcare. Addressing these challenges is essential for improving the welfare of reptiles and promoting responsible pet ownership. To enhance reptile welfare, several key recommendations emerge from our research, such as (i) to conduct awareness campaigns and educational programs to inform the public and reptile buyers/keepers about the specific needs and requirements of reptiles as pets, emphasizing responsible reptile ownership, proper care, and the potential consequences of improper care; (ii) to provide specialized training to veterinarians on reptile medicine and husbandry to diagnose and treat reptile-related health issues effectively; (iii) to regulate reptile breeding and trade to prevent overproduction and ensure that animals are not subject to unsustainable and inhumane practices; (iv) to collaborate with authorities and stakeholders to establish regulations, enforcements, and standards for reptile husbandry that prioritize welfare, based on scientific evidence; (v) to collaborate with conservation organizations and raise awareness about the threats faced by wild reptile populations in order to foster a deeper appreciation for reptiles and their importance in the ecosystem; and (vi) to conduct further research on reptile behavior, ecology, biology, and welfare to improve the understanding of these animals' needs and preferences. Ultimately, by adopting these suggestions and working in tandem with stakeholders, including pet keepers, veterinarians, policymakers, and animal welfare organizations, significant strides in enhancing the welfare of reptiles kept as pets in Brazil can be made.

## Data Availability

The datasets used and/or analysed during the current study are available from the corresponding author upon reasonable request.

## Abbreviations

US$: United States dollar
IBAMA: Brazilian Institute of Environment and Renewable Natural Resources
SD: Standard deviation
UV: Ultraviolet
